# Enhancing *Rubisco* gene expression and metabolites accumulation for better plant growth in *Ficus deltoidea* under drought stress using hydrogen peroxide

**DOI:** 10.3389/fpls.2022.965765

**Published:** 2022-09-30

**Authors:** Mohammad Moneruzzaman Khandaker, Rosnah Jamaludin, Ali Majrashi, Zalilawati Mat Rashid, Sarker Mohammad Rezaul Karim, Hatim M. Al-Yasi, Noor Afiza Badaluddin, Mekhled Mutiran Alenazi, Khamsah Suryati Mohd

**Affiliations:** ^1^School of Agriculture Science and Biotechnology, Faculty of Bioresources and Food Industry, Universiti Sultan Zainal Abidin, Terengganu, Malaysia; ^2^Department of Biology, College of Science, Taif University, Taif, Saudi Arabia; ^3^School of Food Industry, Faculty of Bioresources and Food Industry, Universiti Sultan Zainal Abidin, Terengganu, Malaysia; ^4^Department of Crop Science, Faculty of Agriculture, Universiti Putra Malaysia, Seri Kembangan, Malaysia; ^5^Plant Production Department, College of Food and Agricultural Sciences, King Saud University, Riyadh, Saudi Arabia

**Keywords:** *Ficus deltoidea*, H_2_O_2_, drought, growth, physiology, *Rubisco*, metabolites

## Abstract

Growth improvement of the medicinal plant, *Ficus deltoidea* (Mas Cotek) under drought conditions is a vital issue in Malaysia since it is a slow-growing plant and disposed to leaf damage under the stresses of drought. Therefore, investigation was done to examine the outcomes of hydrogen peroxide (H_2_O_2_) application on *Rubisco* gene expression and metabolites accumulation of stressed *F. deltoidea* plants, and thereby to record the changes in leaf histology, photosynthesis, biochemical properties, and the growth of the plant. H_2_O_2_ at the rates of 0, 5, 10, 15, and 20 mM were foliar sprayed biweekly on the drought stressed plants using a hand sprayer. The application of 20 mM H_2_O_2_ amplified leaf number, tallness, stomatal conductance, and photosynthetic yield by 143, 24, 88, and 18%, respectively, over the control plant. A reduced transpiration rate and improved chlorophyll fluorescence were also noted in H_2_O_2_-treated plants. The treatment produced a greater amount of chlorophyll *a*, total phenols, total flavonoids, sugar content, and antioxidant activities by 1.61-, 1.30-, 1.98-, 1.92-, and 1.53-fold, respectively. Application of 15 mM H_2_O_2_ enhanced net photosynthetic rate and internal CO_2_ concentrations by 1.05- and 1.25-fold, respectively. Additionally, H_2_O_2_ treatments promoted stomatal closure, increased stomata size, the number of stomata, improved vein structure, and reduced the damage of the leaf margin and mesophyll cells of drought stressed plants. The application of H_2_O_2_ also accumulated significantly higher contents of sodium (Na^+^), calcium (Ca^2+^), potassium (K^+^), magnesium (Mg^+^), and iron (Fe^2+^) in stressed plants. Although the amount of Arsenic (As^+^) and Antimony (Sb^3+^) increased to some extent, the increases were not at a toxic level. The use of H_2_O_2_ enhanced the *Rubisco* gene expression to a greater level and the ratio of *Rubisco* expression increased up to 16-fold. Finally, thirteen (13) identified and five (5) unmatched volatile compounds with a quality score above 70% were identified by gas chromatography-mass spectrometry (GCMS). The GCMS analysis showed that the foliar application of H_2_O_2_ accumulates a higher percentage of volatile components in plants which helps to mitigate the negative effects of drought stress. It is concluded that under drought stressed conditions the *F. deltoidea* plants should be treated with 10–15 mM of H_2_O_2_ twice a week to improve leaf histology, photosynthesis, the level of *Rubisco* gene expression and volatile compounds accumulation, and plant growth and development.

## Introduction

An important medicinal plant in Malaysia, *Ficus deltoidea* (Mas Cotek) is a C3 plant of the Moraceae family that can be found in Malaysia, Indonesia, Thailand, The Philippines, South Asia, and Africa (Starr et al., 2003; Hasham et al., 2013; Badron et al., 2014). The plant is grown as a house plant in cooler regions and is also cultivated in various parts of the world in abundance along the peat soil, beaches, and forest areas up to 3,000 m above sea level (Starr et al., 2003; Musa, 2005). It is used in traditional medicinal practices due to its various medical properties and functions. It is effective for healing, is used as a palliative agent, antinociceptive, antioxidant, anti-proliferative, and as preventive medicine which treats postpartum depression and assists in contracting and healing the muscles of the uterus and vaginal canal (Fasihuddin and Din, 2002). Tea from the leaves of *F. deltoidea* contain a considerable amount of magnesium, manganese, potassium, sodium, iron, and zinc, which are used to fulfill human daily nutrient requirements (Nihayah et al., 2012). *F. deltoidea* extracts are used to reduce diabetes complications, increase HDL levels, decrease LDL levels, improve sex life, assist the contraction of the vagina after delivery in addition to having anti-cancer and antimicrobial properties (Aris et al., 2009; Dramant et al., 2012). The plant contains phenols, flavonoids, tannins, triterpenoids and proanthocyanins (Aris et al., 2009; Nazarni et al., 2018). All these phenols and flavonoids display high antioxidant properties, with bioactive compounds vitexin and isovitexin from *F. deltoidea* displaying α-glucosidase inhibition (Nazarni et al., 2018; Nurdiana et al., 2018).

Currently the pharmaceutical industry uses plant-based sources to produce bioactive compounds and secondary metabolites. *F. deltoidea* as a medicinal herb is gaining popularity in local markets for the preparation of herbal tea and capsules (Misbah et al., 2013). This is a slow growing medicinal herb with low photosynthetic capacity since direct sunlight does not enhance its growth resulting in low biomass accumulation. The biomass accumulation in plants is very important for its commercialization. This herb also cannot bear drought stress as it needs plenty of water (6–8% soil moisture) to ensure its optimum growth and development. *F. deltoidea* plant is prone to an unexpected loss of leaves under drought conditions. Drought stress reduces cell division and enlargement, root differentiation, leaf area expansion, plant height, altered stomatal opening and closing, water use efficiency, and decreased plant yield and quality (Kumawat and Sharma, 2018). Due to the closing of the stomata, the activity of photosynthesis decreases, increasing production of reactive oxygen species (ROS), inducing membrane injury and altered function of several enzymes which are associated with ATP synthesis (Kumawat and Sharma, 2018; Sharma et al., 2020). To overcome all these problems in the *F. deltoidea* plant, this study proposes to use H_2_O_2_ to increase photosynthetic capacity, nutrients absorption, improve leaf histology, metabolites accumulation, and improve drought tolerance.

Drought stresses create interruption in plant growth, reduce plant productivity, and in severe cases, lead to plant death. The plant under drought stress produces signals for new structural and metabolic capabilities through gene expression for immediate survival and acclimatization (Bohnert and Sheveleva, 1998). Hydrogen peroxide (H_2_O_2_) may act as a messenger molecule to adapt signaling which triggers anti-susceptibility under abiotic stresses when it orchestrates a programmed cell death (Cheeseman, 2007; Ismail et al., 2015). Kar (2011) reported that the drought stress generates reactive oxygen species (ROS) leading to cellular damage. But H_2_O_2_ is a highly stable ROS at optimum concentration, which acts as a second messenger and sends signals to molecules for the promotion of plant growth, enhancement of photosynthetic activity, and increase in sugar content (Ozaki et al., 2009; Khandaker et al., 2012). Ozaki et al. (2009) also stated that H_2_O_2_ (<50 mM concentration) produced stimulatory effects on plant growth and development of melon plants. A higher concentration of H_2_O_2_ initiates programmed cell death, damage chloroplast and the photosynthesis process, however, findings of Orabi et al. (2015) determined that a lower level of H_2_O_2_ stimulated growth regulators (auxins, gibberellin, and ABA) and enzyme activity. The foliar application of H_2_O_2_ maintains the leaf water content in soybean plants and enables them to avoid drought stress (Ishibashi et al., 2011). Many physiological processes in plants are controlled by H_2_O_2_ such as ABA-induced stomatal opening and closing, stomatal movement, hypersensitive responses, gene expression, programmed cell death, etc. (Neill et al., 2002).

H_2_O_2_ is produced in different enzymatic reactions in plants, which is transported through liquid flow in the plant body. It acts as a growth regulator in several selected plants. It was used in this study as the chemical promoter to overcome the early phase slow growth problem of *F. deltoidea* and to counteract the drought stress effects introduced to *F. deltoidea* plants. Attention was given through investigation to the regulatory effects of H_2_O_2_ in *Rubisco* (*rbcL*) gene expression and metabolite accumulation in *F. deltoidea* under drought stressed conditions to improve biochemical properties, leaf histology, photosynthesis, and plant growth. It was proposed that under drought stress conditions, foliar spraying of H_2_O_2_ could modulate the plant's growth and could mitigate the unfriendly effects of drought in this experimental plant, *F. deltoidea*. The findings of this study will be beneficial in mitigating the negative effects of drought stress in *F. deltoidea* as well as other medicinal herb plants.

## Materials and methods

### Experimental site and preparation of experimental plants

The study was conducted at Universiti Sultan Zainal Abidin, Terengganu, Malaysia during the period from December 2014 to February 2020. The experimental plant *Ficus deltoidea* var. *deltoidea* (FD 156) was collected from Kampung Sungai Nibong, Batu Pahat, Malaysia. The plants were propagated first in the university farm using stem cutting 8.0 inches in size and were used later for treatment application. For preparation of experimental plants, 25 stem cuttings were transplanted into an prepared planting media, each polybag contained 15 kg planting media. Cocoa peat, paddy husk, and perlite were mixed in the ratio of 2:1:1 to prepare the media. Side dressing with 2.5 g of NPK fertilizer (10:10:20) was done once a month. To simulate drought stress conditions in all experimental plants, less water (about 1.5 liters of water for 25 plants per spray) was applied three times per week using a watering can maintaining the moisture content at 40% of field capacity. One hundred eighty (180) ml of water was applied per plant to maintain drought stress of the experimental plants. The experimental plants were grown under a hoop house which provided the plants with direct sunlight and an average temperature about 30–35°C. The maximum PAR was between 300–700 μEm^−2^s^−1^ and relative humidity was 30–60% (**Figure 2A**).

### Treatments application

The plants were sprayed twice a week manually with 10 ml of hydrogen peroxide (H_2_O_2_) at the concentrations of 5, 10, 15, 20 mM and 0 (water as control). The H_2_O_2_ concentration was selected based on previous studies reported by Ozaki et al. (2009) and Khandaker et al. (2012). The spraying was done at the dry leaf stage around 11:00 am, from the vegetative to flowering stage completing 15 foliar sprays during the experimental period. The treatments were applied following a completely randomized design (CRD) with five replications.

### Data collection

#### Growth and physiological parameters

The number of leaves, the tallness of the plant, and the leaf area were recorded. A Leaf Area Meter (Model Portable Laser CI-202, CID Bio-science, USA) was used to quantify leaf area. A measuring tape was used to measure plant height (from base of plant to top of youngest shoot). The SPAD 502 Plus Meter of Konica Minolta; Opti-science, USA was used to record chlorophyll content. A Handy PEA meter (Hansatech-Instrument, UK) was used to record the data on chlorophyll fluorescence in three levels, F0, Fv, and Fv/Fm on sunny days, once a month. The CI-340 handheld Photosynthesis System (Bio-Science, USA) was used to record other physiological parameters, e.g., net photosynthetic rate (*Pn*), transpiration rate (*tr*), internal CO_2_ concentration, and stomatal conductance (g_s_w). As per Khandaker et al. (2012), growth and physiological parameters of treated and untreated plants were recorded eight (8) times and, for each time, data was recorded 2 days after the treatment application. A series of experiments were carried out for the repeated measurements of physiological and growth parameters, nutrient elements and biochemical analysis, gene expression studies, and major compound identifications from leaf extracts of drought stressed plants.

#### Biochemical properties and mineral nutrient analysis

First, the methanolic extract was developed using treated and untreated leaves of *F. deltoidea* for biochemical analysis. Harvested leaves (3 days after the treatment application) were dried for 3 days at 45°C in a dryer. The dried leaves were ground properly in a grinding machine and 10 g of leaf powder were soaked in 70% methanol extract for 3 days and then transferred to an orbitary shaker for mixing. Filtration of the contents was done using Whatsman No. 1 filter paper. The crude methanolic extract was prepared by evaporating the filtrate into a rotary evaporator.

Dubois et al. (1956) method was referred to when measuring the sugar content in the crude methanolic extract. The standard method of Singleton and Rossi (1965) was used to estimate the total phenolic content of the extract. Gallic acid, as a standard chemical was used to express the phenolic content as mg/GAE/g [milligram gallic acid equivalents (GAE) per gram] of dry extract. The Aluminum Chloride colorimetric analysis (Chang et al., 2002) was used to determine the total flavonoid content as mg/QE/g [milligram quercetin equivalents (QE) per gram] dry weight since quercetin was used as a standard chemical. The antioxidant properties of the plants were ascertained by carrying an ABTS assay following the guidelines of the ABTS kit's manual (Sigma-aldrich). The formulae of Arnon (1949) were used to determine the contents of chlorophyll *a, b* and carotenoid. The leaves and syconium of treated and untreated plants were collected at 1 day after the treatment application for mineral nutrients analysis. The Neutron Activation Analysis (NAA) method was used to analyze the mineral accumulation in leaves and syconium, which was described by Nashriyah et al. (2006). The Sodium (Na^+^), Potassium (K^+^), Calcium (Ca^2+^), Magnesium (Mg^+^), Iron (Fe^2+^), Arsenic (As^+^), and Antimoni (Sb^3+^) were analyzed at the Malaysian Nuclear Agency, Bangi, Kajang, Selangor.

### Leaf histology

A histological examination was carried out at the Electron Microscope Scanning laboratory, Institute of Oceanography and Environment (INOS), Universiti Malaysia Terengganu (UMT), Terengganu with the help of a Scanning Electron Microscopy (SEM) (Model JEOL JSm-636OLA) as per methodology of Rohini et al. (2016). The prepared samples were arranged accordingly on the stub for gold coating and the samples were ready to be observed under the SEM. The parts of the leaves that were focused on in this study were the leaf cross section, leaf margin, stomata at lower and upper surfaces of the leaves, as well as stomatal size, and leaf vascular bundle.

### *Rubisco* gene expression

The RNA extraction was done according to the procedure described by Wang et al. (2015) in the manual of SV RNA Isolation System kit (Promega) and the centrifugation was used at 12,000–14,000 rpm. Yield of RNA obtained was determined by using a Spectrophotometer at 260 nm wavelength. The manual of Takara Prime Script 1st strand cDNA synthesis kit was consulted to synthesize the cDNA. The reaction tubes were incubated in heat-block at 70°C for 15 mins to inactivate the reverse transcriptase before doing the PCR and amplification. Aliquots of the single strand cDNA of the treated plants either at 0, 15, or 20 mM were subjected to quantitative RT-PCR analysis on the *rbcL* gene. The expression of the *Co-GAPDH* (glyceraldehydes-3-phosphate-dehydrogenase) gene was considered as the internal control. Using a Rotor-Gene 6000 Series Software 1.7 (Build 65), the expression data were evaluated.

### Gas chromatography-mass spectrometry

Agilent Technologies 7890A/5975C MSD Gas Chromatograph Mass spectrometry system, equipped with HP-5MS Ultra inert capillary column (30 m × 0.25 mm inner diameter, 0.25 μm film thickness) was used to identify the major compounds in the leaf extracts of H_2_O_2_ treated and untreated *F. deltoidea*. Helium was used as the carrier gas and conditioned at 8.2317 psi pressure with a flow rate at 1 ml min^−1^. The samples were injected into the GCMS system with the injector and transfer line temperature conditioned at 250°C. The oven temperature was programmed at 60°C (10 min), 60°C to 180°C at the rate of 3°C/min and 180°C (15 min). The sample was injected into a split mode of front inlet with split ratio 50:1. Compounds present in the samples with quality of the probability score above 70% were selected and identified using the Wiley7Nist05.L and HPCH2205.L Mass Spectral Library.

### Statistical analysis

A completely randomized design was followed to assign the treatments replicated five times. The SPSS-17 statistical software was referred to analyze the data as per the procedure of one-way ANOVA to know the significant difference between the parameters and the Tukey's test (HSD) was used to compare different concentrations of H_2_O_2_ in their effects on the parameters studied at *p* = 0.05.

## Results

### Impacts of H_2_O_2_ on growth and physiological parameters

The regulatory effects of various concentrations of liquid H_2_O_2_ and water were observed on drought-stressed *Ficus deltoidea* plants. The number of leaves were significantly influenced by the treatments, with the highest number of leaves recorded in the 20 mM treated plants. The plant height was 143% higher with the 20 mM H_2_O_2_ treatment as compared to the control plants, meaning that H_2_O_2_ application stimulated plant height of *F. deltoidea* in drought conditions although the differences were insignificant (Table 1). The leaf area was also not affected significantly with different treatments of H_2_O_2_.

**Table 1:** The effects of H_2_O_2_ treatment on plant growth and photosynthetic characteristics of drought stressed *F. deltoidea* plants.

**Treat** (**mM H_2_O_2_)**	**Number of leaves**	**Plant height (cm)**	**Leaf area (cm^2^)**	**Photosynthetic rate (μmol/m^2^ /s)**	**Transpiration rate** **(mmol/m^2^ /s)**	**Stomatal conductance** **(mmol/m^2^ /s)**
0	234.05 ± 24.5a	47.05 ± 2.09a	6.02 ± 0.90a	2.64 ± 0.54a	0.30 ± 0.05a	32.92 ± 1.85a
5	297.07 ± 35.1a	47.70 ± 2.95a	6.61 ± 0.76a	2.68 ± 0.62a	0.25 ± 0.04a	39.95 ± 2.03b
10	309.47 ± 45.6a	51.33 ± 2.81a	6.78 ± 0.85a	2.69 ± 0.43a	0.25 ± 0.06a	45.32 ± 2.55c
15	458.03 ± 65.9b	57.10 ± 3.78a	6.85 ± 0.79a	2.73 ± 0.40b	0.22 ± 0.06a	61.76 ± 2.90d
20	568.43 ± 81.9c	58.45 ± 3.64a	7.32 ± 0.98a	2.76 ± 0.55b	0.20 ± 0.04a	62.07 ± 3.07e

Mean values in the same column with the different letters are significantly different according to Tukey's HSD test at p value 0.05.

The net photosynthetic rate was significantly higher in all H_2_O_2_ treatments under drought stress. The application of H_2_O_2_ at 15- and 20-mM concentrations produced higher photosynthesis rates which were 1.05- and 1.03-fold higher than the control plant. The lowest photosynthesis rate was found in the untreated plants at 2.64 μmolCO_2_/m^2^/s. Therefore, the results indicated that H_2_O_2_ treatment could be a factor that promotes better photosynthetic rate in drought conditions (Table 1). For stressed plants, the highest transpiration rate was observed in the control plants with 0.3 μmolCO_2_/m^2^/s and the lowest transpiration rates were noted in the treatments of 20 and 15 mM H_2_O_2_. These were 33% and 27% lower than the control plants. These results signify that stomatal conductance was influenced differently between the treated and untreated plants under drought stressed conditions. Therefore, application of H_2_O_2_ is a method to reduce transpiration rate in stressed plants under drought conditions (Table 1).

The lowest stomatal conductance was observed in untreated plants at 32 mmol CO_2_/m^2^/s (Table 1) but it was 1.88- and 1.87-fold higher in the 20 mM and 15 mM H_2_O_2_ treated plants, respectively (Table 1). The internal CO_2_ was also influenced by different treatments of H_2_O_2_ (Table 2). The lowest internal CO_2_ was detected in control plants at 618 ppm, ambient CO_2_. Plants under the treatments of 15 mM and 20 mM H_2_O_2_ had significantly higher internal CO_2_ levels, which were 1.24- and 1.28-fold respectively, greater than the control plants. Leaf chlorophyll content (estimated by SPAD value) increased by at least 10% due to the different treatments of H_2_O_2_ (Table 2).

**Table 2:** The effects of H_2_O_2_ treatment on leaf chlorophyll content (SPAD) and chlorophyll fluorescence of *F. deltoidea* plants under drought conditions.

**Treat** **(mM H_2_O_2_)**	**Internal CO_2_ (ppm)**	**Chlo. content (SPAD)**	**Lower chlo fluo. (F0)**	**Higher chlo fluo. (Fm)**	**Variable fluo. (Fv)**	**Photosynthetic yield (Fv/Fm)**
0	63.40 ± 4.54b	51.90 ± 4.98a	702 ± 30.2a	2198 ± 209a	1496.1 ± 245c	0.61 ± 0.05a
5	57.45 ± 3.87ab	54.07 ± 2.88ab	707 ± 22.5a	2271 ± 225ab	1563.8 ± 215b	0.62 ± 0.04a
10	52.85 ± 3.90ab	55.51 ± 1.77ab	710 ± 35.0a	2533 ± 190ab	1828.3 ± 157a	0.69 ± 0.03ab
15	91.77 ± 4.80c	56.99 ± 2.69ab	728 ± 8.88a	2538 ± 225ab	1809.9 ± 150a	0.71 ± 0.03ab
20	47.84 ± 3.05a	57.82 ± 2.63b	721 ± 39.3a	2655 ± 259b	1934.19 ± 169a	0.72 ± 0.02b

Mean values in the same column with the different letters are significantly different according to Tukey's HSD test at p value 0.05.

**Figure 1:**
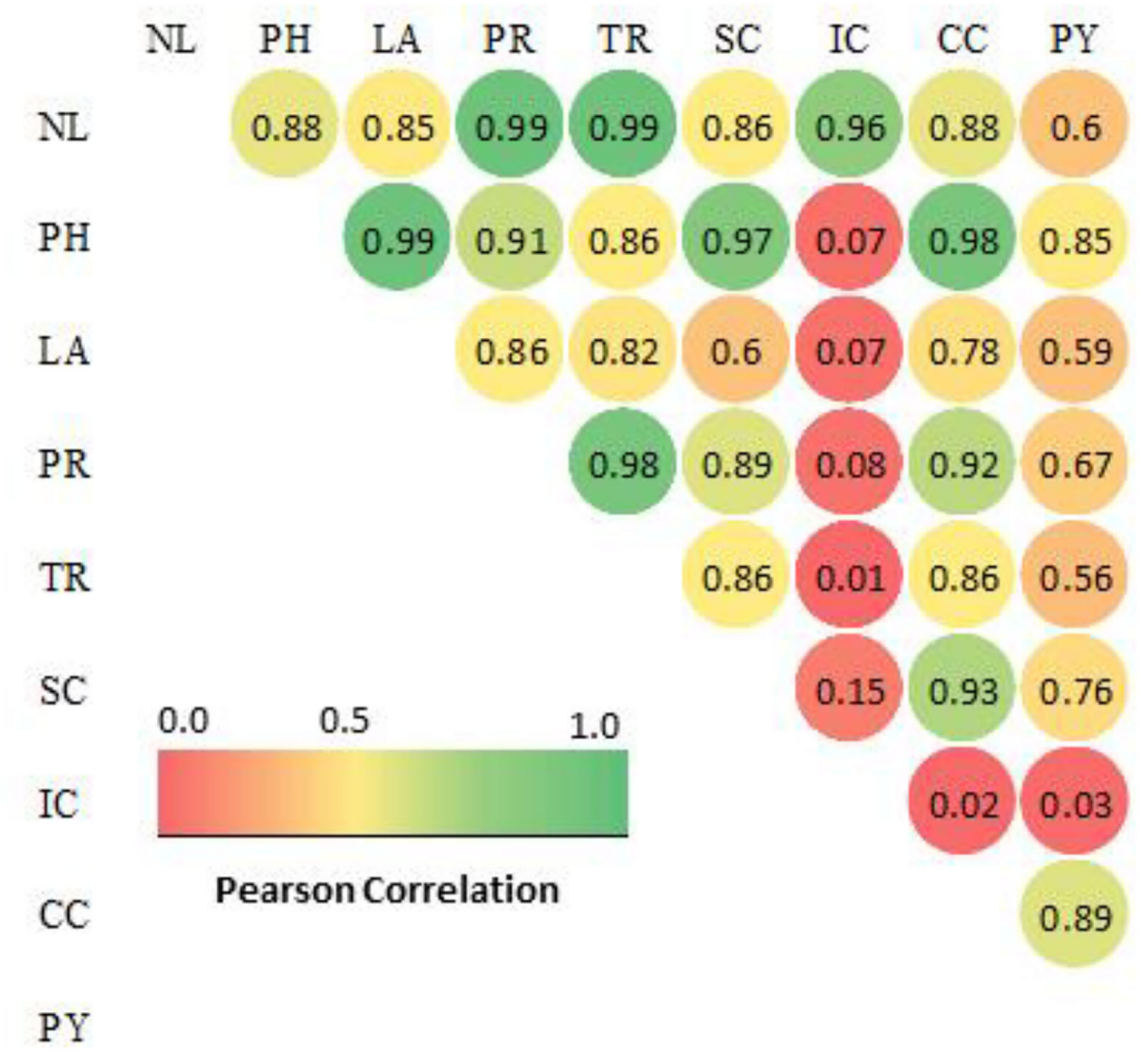
Correlation matrix of plant growth and physiological characteristics in H_2_O_2_ treated *F. deltoidea* plants under drought conditions[[Inline Image]]. The labels of data are similar in Tables 1, 2. Nl, number of leaves; PH, plant height; LA, leaf area; PR, photosynthetic rates; TR, transpiration rates; SC, stomatal conductance; IC, internal CO_2_ concentration; CC, chlorophyll content (SPAD); PY, photosynthetic yield (Fv/Fm). The colored gradient legends represent coefficients of correlation r values from 1.0 (green), 0.5 (yellow) and 0.0 (red). The value >0.9 a strong correlation, >0.8 a fair strong correlation and >0.6 a moderately strong correlation at *p value 0.05*.

The chlorophyll fluorescence value (F_0_) and relative variable fluorescence (Fv) were significantly increased when H_2_O_2_ was applied to drought-stressed *F. deltoidea* plants. Plants treated with 20 mM of H_2_O_2_ produced a 20% higher Fm value compared to the control group. The photosynthetic yield increased 1.18-fold due to the application of 20 mM H_2_O_2_ (Table 2). The growth and plant physiological parameters of H_2_O_2_ treated plants were significantly positively correlated to each other except for the internal CO_2_ concentration (Figure 1). Additionally, the results show that the number of leaves, plant height, and leaf area were positively correlated with net photosynthetic rate, transpiration rate, stomatal conductance, chlorophyll content, and photosynthetic yield of H_2_O_2_ treated *F. deltoidea* plants under drought stressed conditions (Figure 1).

### Effects of H_2_O_2_ on biochemical properties

The chlorophyll content of leaves increased in the treated plants (Table 3) and this was highest for the treatment of 20 mM. Plants treated with 20 mM and 15 mM H_2_O_2_ yielded 1.67- and 1.59- fold higher chlorophyll *b* content (Table 3). The lowest carotenoid content was recorded in fresh leaves extract in control (22% lower) compared to the 20 mM treated plants (Table 3). Therefore, application of H_2_O_2_ on drought-stressed Mas Cotek plants had a significant effect to increase carotenoid content of the plants (Table 3).

**Table 3:** The effects of H_2_O_2_ on chlorophyll content, biochemical properties, and antioxidant activities of drought stressed *Ficus deltoidea* plants.

**Treat** **(H_2_O_2_) (mM)**	**Chlorophyll a** **(mg/L FE)**	**Chlorophyll b** **(mg/L FE)**	**Carotenoid (μg/g FE)**	**TPC** **(mgGAE/g DE)**	**TFC** **(mgQE/g DE)**	**TSC** **(GE μmol/g DE)**	**ABTS** **(mMTrolox/g DE)**
0	11.54 ± 0.01a	4.61 ± 0.28a	1.66 ± 0.04a	99 ± 10a	12.2 ± 0.2a	2.3 ± 0.02a	2.24 ± 0.28a
5	13.81 ± 0.13b	6.15± 0.13b	1.32 ± 0.001a	101 ±1.2a	16.32 ± 0.29b	2.71 ± 0.01b	2.44 ± 0.29a
10	14.75 ± 0.06c	6.93 ± 0.3bc	1.71 ± 0.02b	117 ± 0.88b	19.69 ± 0.1c	2.85 ± 0.01b	2.84 ± 0.20a
15	18.1 ± 0.06d	7.33 ± 0.13c	2.03 ± 0.01c	125 ± 1.53c	21.42 ± 0.15d	4.33 ± 0.06c	3.34 ± 0.18a
20	18.61 ± 0.09e	7.72 ± 0.01c	2.13 ± 0.03c	129 ± 1.33c	24.23 ± 0.22e	4.43 ± 0.03c	3.44 ± 0.15a

Means (±SE) within the same column followed by the same letter do not differ significantly according to the Tukey HSD test at p value 0.05.

The content of total phenolic (TPC) and total flavonoid (TFC) also increased with the treatment of H_2_O_2_ (Table 3). An amount of 1.30- and 1.26- fold higher TPC and TFC content were produced with the application of 15 mM and 20 mM H_2_O_2_ to the drought-stressed plants (Table 3). The production of TFC under the treatment of 20 mM H_2_O_2_ was 98% higher than in the control plants (Table 3). The lowest TFC was determined in control plants with 12.2 mg quercetin equivalent (QE) per gram of dry leaf extract. Similarly, the total sugar content (TSC) also increased in the leaf extract of treated plants of *F. deltoidea* (Table 3). The lowest TSC was determined in untreated plants at 2.1 μmol glucose equivalent (GE) per gram of dried leaf extract. The increment of TSC in 15 mM and 20 mM H_2_O_2_ treated plants was 1.88- and 1.92-fold higher, respectively (Table 3). The results of ABTS assay test indicates an increment of 49 and 54%, respectively, antioxidant activity in a *F. deltoidea* methanolic leaf extract under the treatments of 15 and 20 mM in comparison to quercetin as a positive control. However, the differences between the treatments were immaterial (Table 3).

### Effects of H_2_O_2_ on nutrient uptakes

Mineral uptake and accumulation in the H_2_O_2_-treated plants under stressed conditions was positively influenced. The results of Neutron Activation Analysis (NAA) showed that the application of H_2_O_2_ promoted sodium (Na) intake into the plants. The highest Na content in a leaf was detected in the 15 mM H_2_O_2_ treated plants with 95 μg per gram of dried leaf extract. The lowest Na content was detected in the 20 mM H_2_O_2_ treated plants with a value of 70 μg per gram of dried leaf extract, which was significantly less than the 15 mM treated plants (Table 4). The Na content in the treated *F. deltoidea* syconium was different from the untreated plants. The lowest Na content was observed on control plants with only 260 μg per gram syconium dried extract, meanwhile the highest Na content was detected in the 10 mM H_2_O_2_ treated plants with 810 μg per gram syconium dried extract. Hence, the application of H_2_O_2_ showed stimulative effects on Na uptake by the plants as all treated plants had higher Na content than the control plants (Table 4). On the other hand, the trend of potassium (K) content in *F. deltoidea* leaves was different from the Na content, with the highest found in the control and 10 mM H_2_O_2_ treated plants (2.37 and 2.04% K) (Table 4). The lowest K content in *F. deltoidea* leaves was in the 20 mM H_2_O_2_ treated plants. The opposite pattern was observed in K content of *F. deltoidea* syconium. It was marked that the lowest K content was in the control and 5 mM H_2_O_2_ treated plants, but the highest K content was observed in the plants treated with the highest concentration of H_2_O_2_. Hence, it can be said that H_2_O_2_ promoted K uptake especially in syconium rather than in *F. deltoidea* leaves (Table 4).

**Table 4:** The effects of H_2_O_2_ on macro mineral uptake and accumulation of *F. deltoidea* plants under drought stressed conditions.

**Treat** **(mM)**	**Na (**μ**g/g)**		**K (%)**		**Ca (%)**		**Mg**
	**Leaf**	**Syconium**	**Leaf**	**Syconium**	**Leaf**	**Syconium**	**Leaf**
0	81.3 ± 0.25ab	260 ± 0.18a	2.37 ± 0.22b	3.24 ± 0.23a	1.74 ± 0.12 a	1.34 ± 0.22b	0.59 ± 0.06a
5	85.0 ± 0.18ab	360 ± 0.18a	1.91 ± 0.17a	3.15 ± 0.22a	2.12 ± 0.28 a	0.56 ± 0.19a	0.55 ± 0.18a
10	85.0 ± 0.11ab	810 ± 0.24b	2.04 ± 0.21b	3.98 ± 0.17b	2.44 ± 0.16 b	0.71 ± 0.14a	0.51 ± 0.21a
15	95.0 ± 0.17b	410 ± 0.19ab	1.96 ± 0.16a	3.30 ± 0.19a	2.41 ± 0.16 b	0.74 ± 0.27a	0.64 ± 0.18a
20	70.0 ± 0.25a	290 ± 0.23a	1.90 ± 0.16a	4.12 ± 0.24b	2.26 ± 0.19 a	0.72 ± 0.31a	0.52 ± 0.22a

Mean values in the same column with the different letters are significantly different according to Tukey's HSD test at p value 0.05.

The spraying of H_2_O_2_ produced higher (1.74%) calcium (Ca) content in *F. deltoidea* leaves than in the control plants (1.74%), while the highest amount was determined in the 10 and 15 mM H_2_O_2_-treated plants (2.44 and 2.41%) (Table 4). The opposite trend of Ca content was observed in the syconium. The untreated plants produced the highest Ca content (1.34%) in the syconium while the lowest Ca content was found in the 5 mM H_2_O_2_ treated plants. Generally, calcium content is higher in plant leaves than in the syconium of the plants, but the study indicates different results (Table 4). The same pattern of stimulative effect was observed in magnesium (Mg) uptake in treated plants under drought-stressed conditions. It was determined that the highest Mg content was in the 15 mM H_2_O_2_ treated plants with 0.64 μg per gram of dried leaf extract while the control plants contained a lower Mg content of 0.59 μg per gram of dried leaf extract (Table 4). On the other hand, the control plants had the highest Mg content in *F. deltoidea* syconium with 0.41 μg per gram of syconium dried extract (Table 4). Our results confirmed that the 15 mM H_2_O_2_-treated plants gave positive impacts on Mg uptake in plant leaves, but Mg uptake and accumulation was higher in the syconium of control plants (Table 5).

**Table 5:** The effects of H_2_O_2_ on micro mineral uptake and accumulation of *F. deltoidea* plants under drought stressed conditions.

**Treat (mM)**	**Mg (μg/g)**	**As (**μ**g/g)**		**Sb (**μ**g/g)**		**Fe (**μ**g/g)**	
	**Syconium**	**Leaf**	**Syconium**	**Leaf**	**Syconium**	**Leaf**	**Syconium**
0	0.41 ± 0.21b	0.50 ± 0.18a	0.73 ± 0.11b	0.03 ± 0.01a	0.02 ± 0.06a	60.8 ± 0.19a	59.6 ± 0.10a
5	0.34 ± 0.12a	0.50 ± 0.23a	0.72 ± 0.21b	0.07 ± 0.05a	0.02 ± 0.20a	77.6 ± 0.21a	51.0 ± 0.18a
10	0.26 ± 0.04a	1.2 ± 0.18b	0.34 ± 0.18a	0.10 ± 0.10a	0.14 ± 0.17b	63.3 ± 0.09a	110 ± 0.10b
15	0.30 ± 0.20a	0.78 ± 0.11ab	0.59 ± 0.19b	0.07 ± 0.17a	0.06 ± 0.01a	136 ± 0.21b	94.2 ± 0.23b
20	0.35 ± 0.21a	0.65 ± 0.04a	0.19 ± 0.18a	0.20 ± 0.05b	0.02 ± 0.02a	65.2 ± 0.05a	68.8 ± 0.06a

Mean values in the same column with the different letters are significantly different according to Tukey's HSD test at p value 0.05.

The lowest arsenic (As) content in *F. deltoidea* leaves was detected in the control and 5 mM treated plants with 0.5 μg per gram of dried leaf extract, and the highest As content was noticed in the plants when treated with 10 mM H_2_O_2_ with 1.2 μg per gram of dried leaf extract (Table 5). On the other hand, the higher arsenic content in the syconium were detected in the control and 5 mM H_2_O_2_ treated plants and the lowest As content in the plant syconium was determined in the 20 mM H_2_O_2_ treated plants with only 0.19 μg per gram syconium dried extract. So, it can be said that the exogenous H_2_O_2_ application promoted arsenic accumulation in plant leaves, while it suppressed the As uptake in the plant syconium (Table 5). Antimony (Sb) content was present in *F. deltoidea* leaves only at trace amount. The Sb content in leaves was 6.67-fold higher in 20 mM H_2_O_2_ treated plants compared to the control plants and their differences were statistically significant (Table 5). The same increasing trend of Sb content was found in the syconium. This shows that 10–20 mM H_2_O_2_ were the best concentrations to promote Sb intake in both plant leaves and syconium (Table 5).

The iron (Fe) content in *F. deltoidea* leaves was observed at the lowest level in the control plants with 60.8 μg per gram of dried leaf extract, meanwhile the highest Fe content was detected in the 15 mM H_2_O_2_ treated plants with 136 μg per gram of dried leaf extract, which was significantly greater than the untreated plants (Table 5). In the syconium, the Fe content was also 1.58 times higher in treated plants compared to the control plants and their difference was statistically significant (Table 5). Therefore, H_2_O_2_ at the concentration of 15 mM was the best dose in promoting iron uptake in both plant leaves and syconium under drought stressed conditions (Table 5).

### Regulatory effects of hydrogen peroxide on leaf histology

**Figure 2:**
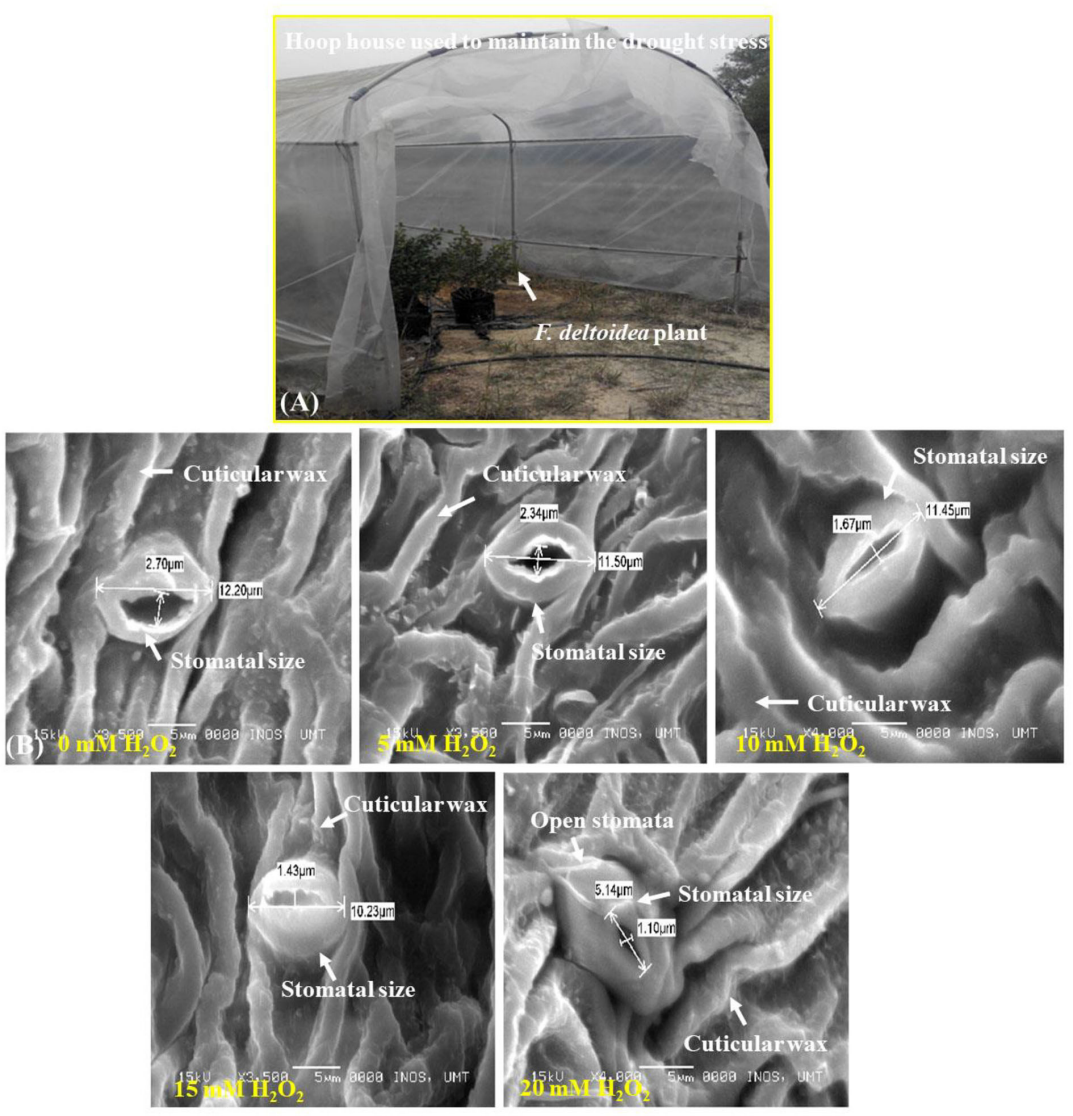
**(A)** A hoop house was used to maintain drought conditions to the experimental plants. **(B)** Scanning Electron Microscopy (SEM) photographs of stomatal closure at lower surface of treated and untreated leaves of *F. deltoidea* plants. Stomatal closure was the widest in control plants whereas the smallest stomatal closure was recorded in the 20 mM H_2_O_2_ treated plants. Deformed stoma and guard cell are visible at the leaf of control plants.

Several parts of leaves were examined under SEM to determine H_2_O_2_'s effect on leaf histology of stressed *F. deltoidea* plants. H_2_O_2_ treatments under drought stress promoted stomatal closure of *F. deltoidea* leaves in a dose-dependent manner. The control treatment (H_2_O_2_ at 0 mM) had the widest leaf stomatal closure which was 2.7 μm. The smallest stomatal closure was observed in the highest H_2_O_2_ treated leaves (20 mM H_2_O_2_) which was 1.1 μm (Figure 2). It could be speculated that the application of exogenous H_2_O_2_ acts as an intermediary to promote guard cell closure under severe drought conditions in the presence of ABA.

**Figure 3:**
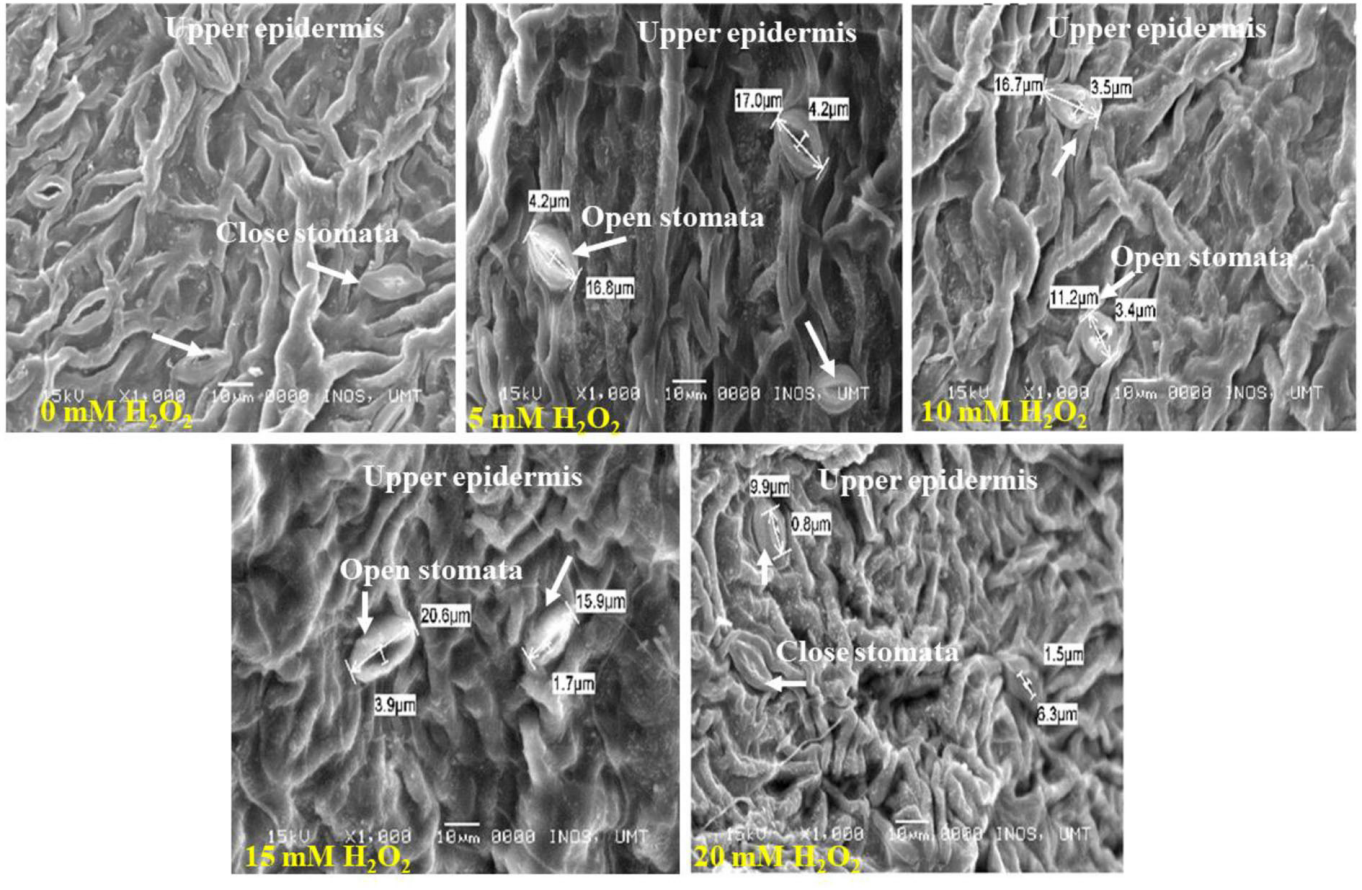
SEM photographs of stomatal opening at leaf's upper surface of H_2_O_2_ treated and untreated plants. Stomata are closed at control treatment, but the stomatal opening or size increased with H_2_O_2_ treatment (5 mM−15 mM H_2_O_2_). Stomatal size starts to decrease again with a higher concentration of H_2_O_2_. Less number of stomata contained at the upper surface of the leaves.

**Figure 4:**
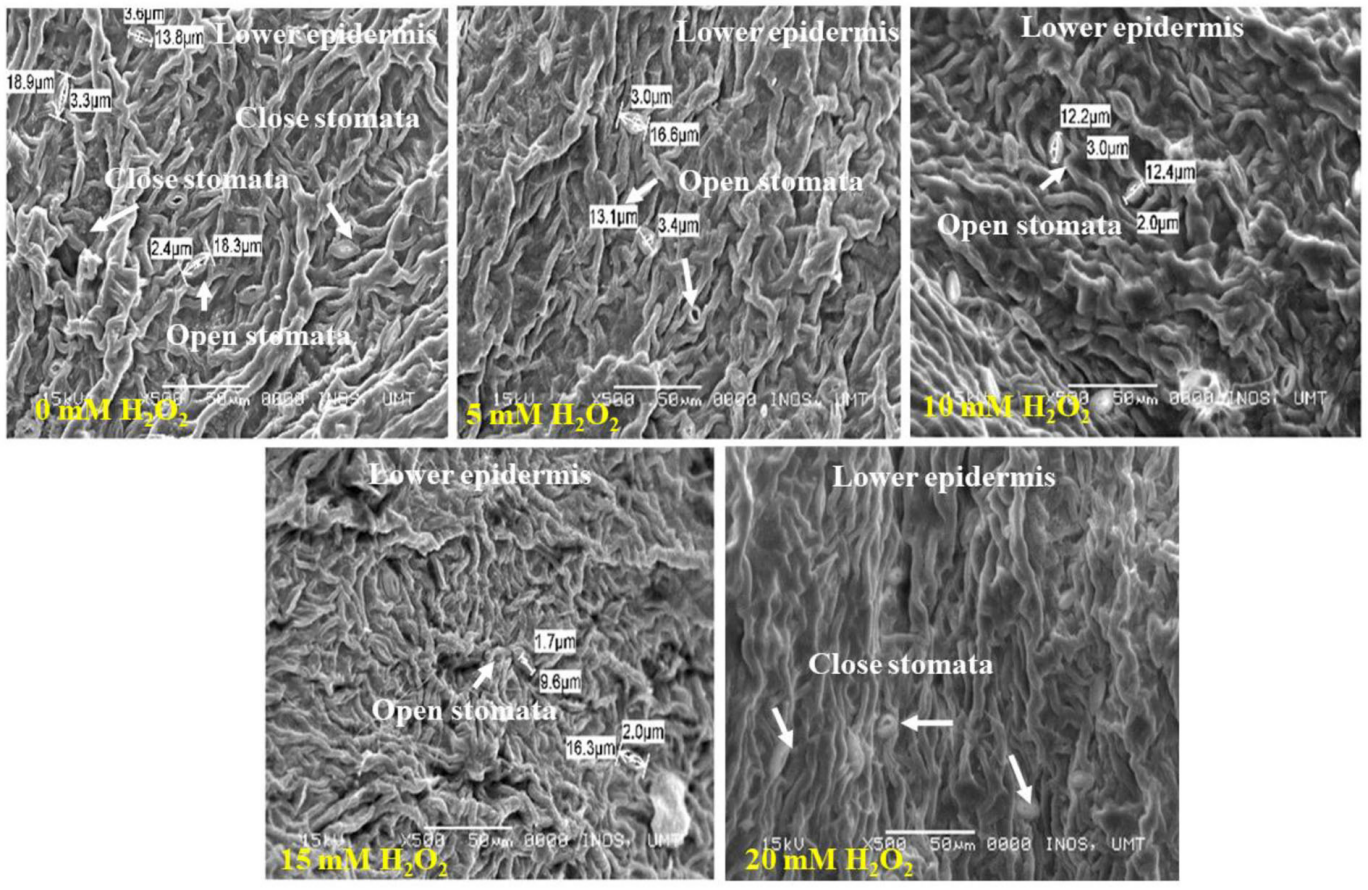
SEM photographs of stomatal closure at lower surface of the *F. deltoidea* leaves. Lower leaf surface contained a higher number of stomata than the upper surface. The smallest stoma size was observed in the 15 mM H_2_O_2_ treatment and stoma size started to decrease with an H_2_O_2_ concentration increase under drought stress.

The application of H_2_O_2_ produced significant changes on stomatal size in the upper and lower surfaces of the leaf (Figures 3, 4). The stomatal opening found on the upper surface was smaller. The control plant had 1.6–3.1 μm of a stomatal opening but the size increased when applied with H_2_O_2_ (Figure 3). Plants treated with 20 mM H_2_O_2_ had the lowest stomatal size of 0.8–1.5, which were significantly different from the untreated plants. Therefore, it is clear that the stomatal opening or closure is stimulated by H_2_O_2_ under stressed field conditions (Figure 3).

**Figure 5:**
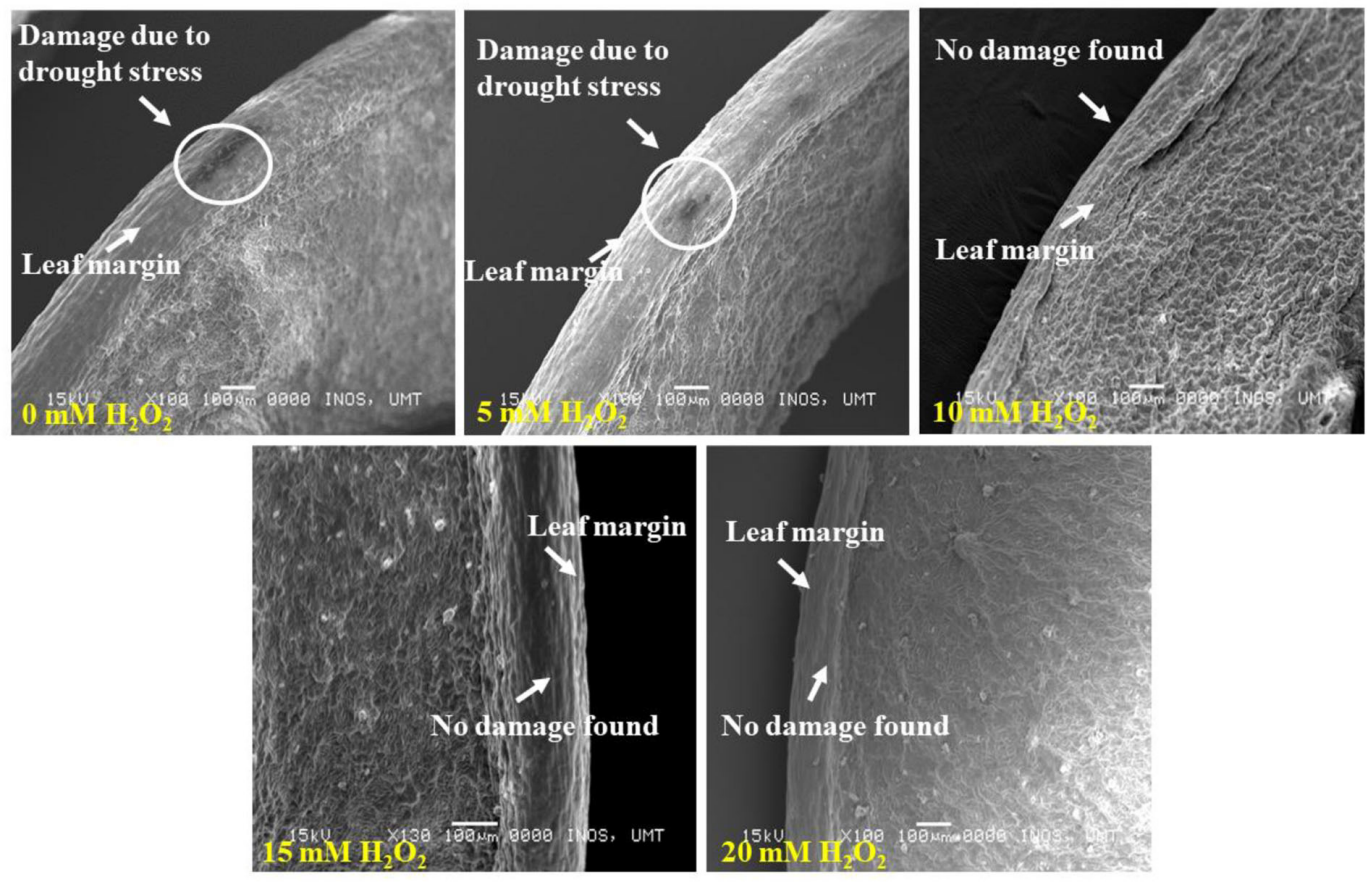
SEM photographs showing the leaf margin of *F. deltoidea* plants. Damaged appeared in the leaf margin of the control and 5 mM H_2_O_2_ treated plants. No damage was found in the 10–20 mM H_2_O_2_ treated plants and it is proven that exogenous H_2_O_2_ can protect leaf structure from oxidative damage induced by drought stress.

The lower leaf surface contained more numbers of stomata, and the largest stomatal size (1.14–4.1 μm) was recorded with the 20 mM H_2_O_2_ treatment (Figure 4). The H_2_O_2_ application also had a stimulatory effect on the stomatal size under stressed conditions. Treated plants with 5 and 10 mM of H_2_O_2_ had stomatal sizes of 3.0–3.4 μm and 2.0–3.0 μm, respectively. The smallest stomatal sizes (1.7–2.0 μm) were observed in the 15 mM H_2_O_2_ treated leaves (Figure 4), with the possibility that 15 mM H_2_O_2_ concentration is the best concentration to promote stomatal closure under drought stressed field conditions. Although slight damage appeared on the leaf margin of the control plants and the plants treated with a low dose, 5 mM H_2_O_2_, under drought-stressed conditions (Figure 5), no damage was spotted on the leaf margin of *F. deltoidea* plants treated with higher doses, e.g., 10, 15, and 20 mM H_2_O_2_. The results confirm that spraying H_2_O_2_ could protect the leaf margin of *F. deltoidea* plants from damage caused by drought stress (Figure 5).

**Figure 6:**
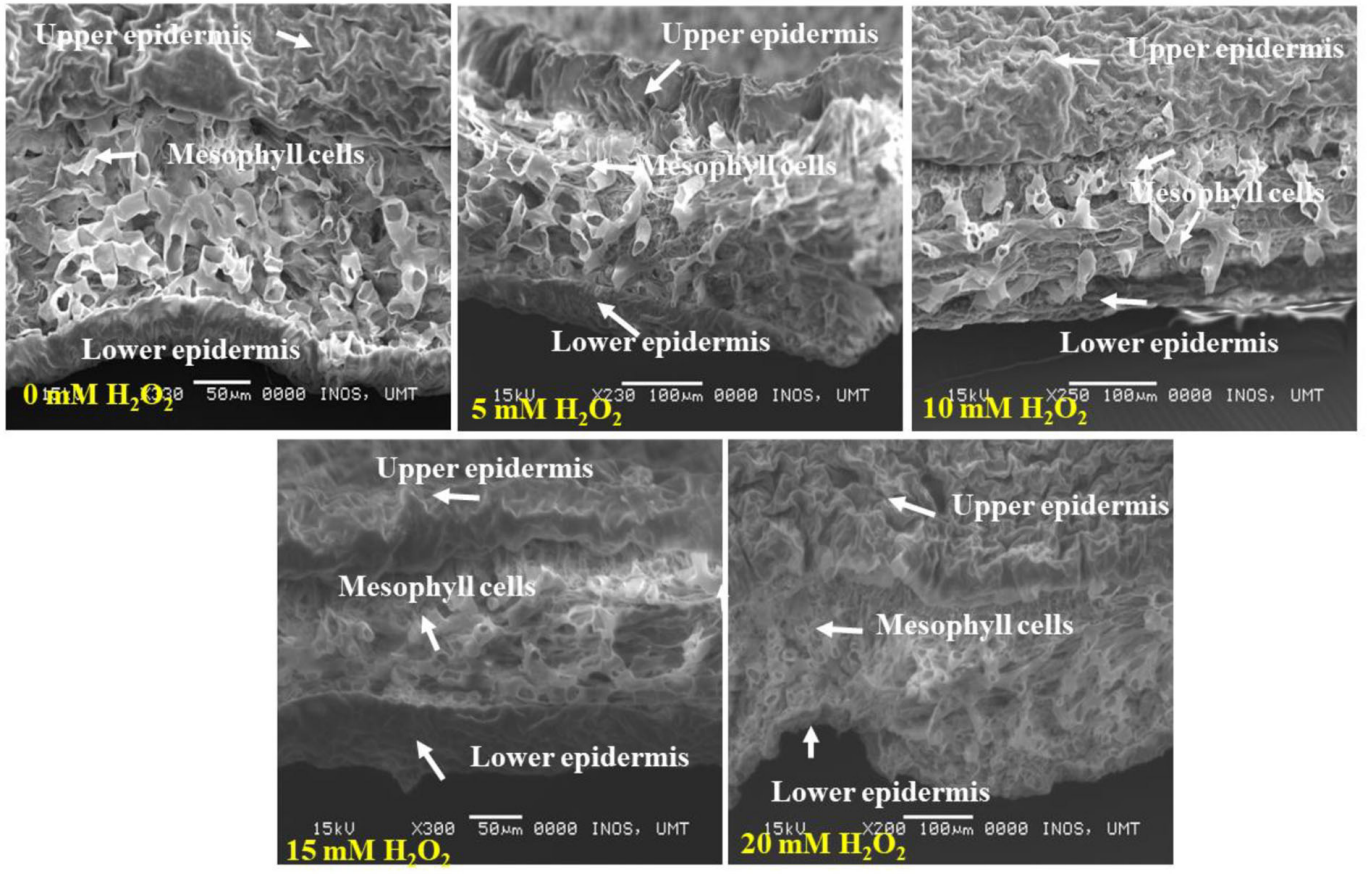
SEM photographs show the cross section of *F. deltoidea* leaves under drought stressed condition. The photographs show upper and lower leaf surfaces, each surface consists of an epidermis layer, cuticular wax, and mesophyll cells. Well-developed mesophyll cells are visible in 5 mM and 10 mM H_2_O_2_ treated plant leaves.

**Figure 7:**
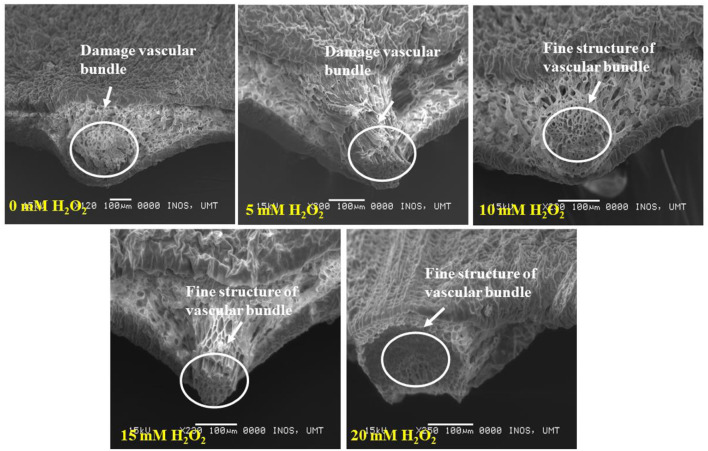
SEM photographs show the cross section of drought stressed *F. deltoidea* leaf veins. Fine structure of the vascular bundle (xylem and phloem tissues) is visible in the 10–20 mM H_2_O_2_ treated plant leaves. Damaged leaf vascular tissues are found in the control and 5 mM H_2_O_2_ treated plants.

Figure 5 demonstrates the cross section of treated and untreated leaves of *F. deltoidea* plants. It was noticed that the application of H_2_O_2_ reduced the damage to mesophyll cells in the stressed plants. Treated leaves with 5, 10, 15, and 20 mM H_2_O_2_ had finer spongy and palisade mesophyll layers than the control plants. This might be related to the function of H_2_O_2_ in alleviating stress in the plants (Figure 6). When the cross section of the leaf vein was examined, a similar kind of positive effect, as noticed in mesophyll cells, was observed. All the treated plants had better vein structure than the untreated plants (Figure 7). It was observed that untreated plants had more or less a compact structure of xylem and phloem, whereas H_2_O_2_, treated plants had more turgid cells of tracheid in xylem and well-developed phloem tissue. It was also detected that the tracheid and vessel's cells in the vascular bundle were well developed and organized in the treated plants (Figure 7).

### Effect of hydrogen peroxide on *Rubisco* gene expression

**Figure 8:**
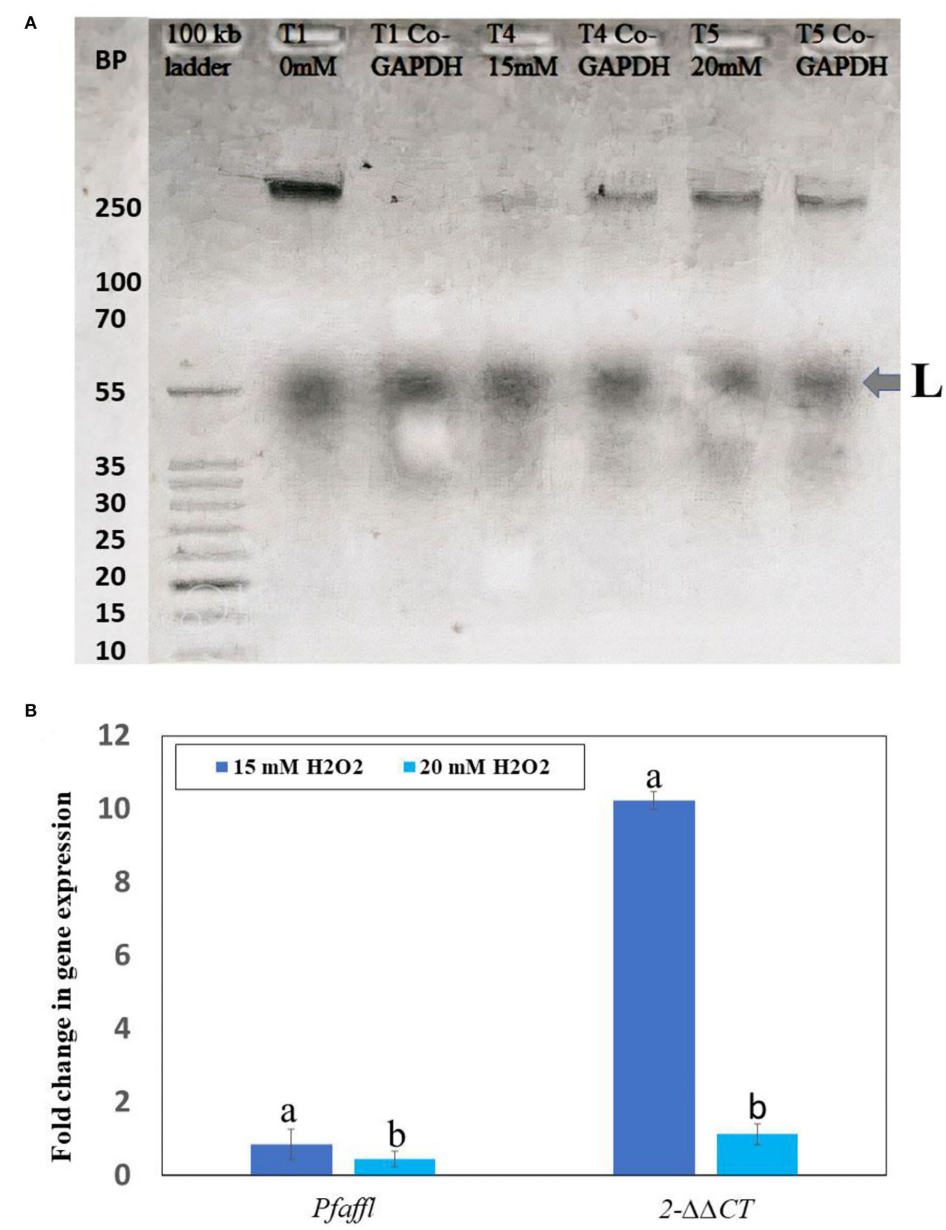
The result of RT-PCR when viewed under gel electrophoresis **(A)**. The *Pfaffi* and *2*^−ΔΔCT^ methods were used to analyze the fold change in *rubisco* (*rbcL*) gene expression in the control, 15 mM and 20 mM H_2_O_2_ treated plants **(B)**. Bars indicate (± S. E.). Means followed by the same lower case letters within the column are not significantly different according to Tukey's HSD-test (n = 3, *p* ≤ *0.05*).

The effects of H_2_O_2_ on *Rubisco* gene expression in *F. deltoidea* plants were more visible in the study. H_2_O_2_ also regulated the *rbcL* gene expression in drought-stressed plants of *F. deltoidea*. In earlier studies, it was reported that the spraying of H_2_O_2_ at the concentrations of 15 and 20 mM produced the highest stimulative effects on a leaf histology, photosynthesis, biochemical properties, and plant growth, under drought-stressed conditions. In this study, 0 (control), 15, and 20 mM H_2_O_2_ treatments allow us to see the H_2_O_2_ effects on the rubisco gene expression and fold change, and determine the best treatment compared to the control. Based on plant growth and physiological performance we selected these three treatments for the *rbcL* gene expression study. The Real-Time PCR (RT-PCR) test confirmed the stimulative effects on the *rbcL* gene expression observed in a gel electrophoresis. Figure 8A displays the presence of the *rbcL* gene in both the leaves treated with 15 mM and 20 mM H_2_O_2_ and also in the leaves of control plants when compared with the housekeeping gene (Co-GAPDH). The fold change of *rbcL* gene was also calculated following *Pfaffl* and *2*^−ΔΔCT^ methods in comparison to 0 mM H_2_O_2_ as the control gene. Quantitative analysis indicated that the *rbcL* gene expression ratio was the highest 0.8649- (*Pfaffl* method) and 10.23-fold higher (*2*^−ΔΔCT^method) in the treatment of 15 mM H_2_O_2_ when compared to the control plants (Figure 8B). However, the plants treated with 20 mM of H_2_O_2_ had a slightly lower fold change of *rbcL* gene in comparison to the 15 mM H_2_O_2_ treatment, but it was still higher than the control. Therefore, application of 15 mM H_2_O_2_ promotes the expression of the *rbcL* gene at a better rate than 20 mM H_2_O_2_ treatments under drought stressed field conditions (Figure 8). The *rbcL* gene fold change was the lowest in the control gene (0 mM H_2_O_2_).

### Gas chromatography-mass spectrometry analysis

**Figure 9:**
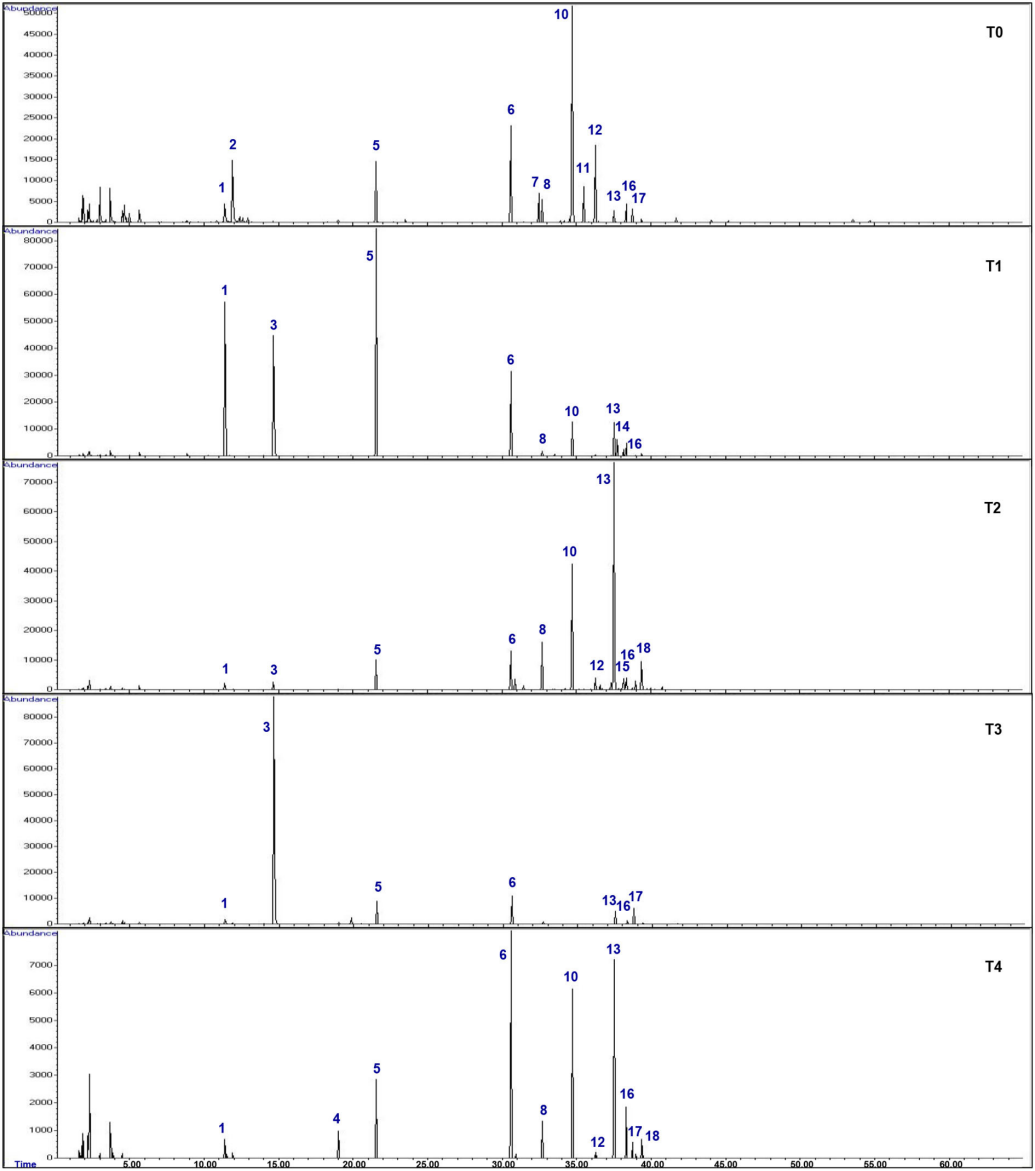
Gas chromatography mass spectrometry (GCMS) total ion chromatograms (TIC) of the leaf extracts of H_2_O_2_ treated and untreated *F. deltoidea* plants under drought stress. Thirteen (13) identified and five (5) unmatched volatile compounds with a quality score above 70% were identified, which are responsible for drought stress mitigation.

GC-MS analysis was carried out to analyze the effects of hydrogen peroxide (H_2_O_2_) treatments (T1, 5 mM; T2, 10 mM; T3, 15 mM; T4, 20 mM) on drought stressed *F. deltoidea* for the major compounds in the leaf extracts compared to the untreated (T0) plants. The retention time (RT) and relative abundance (%) were recorded in this study. The comparison of total ion chromatograms of the control and treated groups (Figure 9) revealed the differences in percentages of 18 volatile compounds in the leaves following *F. deltoidea* treatment with H_2_O_2_. Thirteen (13) identified compounds with a quality score above 70% were identified, while five unmatched compounds were labeled as U1–U5 (Table 6). In this study, the aromatic volatile components detected in the native leaf extracts (T0) were mainly (E)-Caryophyllene (38.88%) at minute (min) 34.72, followed by α-Humulene (12.70%) at min 36.28, while the least amount was (E)-β-Ocimene (0.50%) at min 14.66. Other compounds detected were α-trans-Bergamotene (5.42%), α-Ylangene (3.38%), (E, E)-α-farnesene (2.94%), α-copaene (2.37%), and Germacrene D (1.26%). The volatile components detected in the leaves of the 5 mM H_2_O_2_ (T1) treated plants were mainly (*E*)-β-Ocimene (23.08%) at minute 14.66, followed by Germacrene D (15.86%) at minute 37.52, (*E*)-Caryophyllene (15.36) at minute 34.72, δ-Selinene (6.73%) at minute 37.75, and α-Copaene (1.96%) at minute 32.69, respectively (Table 6). On the other hand, the volatile components detected in the leaves of the 10 mM H_2_O_2_ (T2) treated plants were mainly Germacrene D (32.90%) at minute 37.52, followed by (*E*)-Caryophyllene (20.21%) at minute 34.72, α-Copaene (5.07%) at minute 32.69, δ-Cadinene (3.07%) at minute 39.38, and α-Guaiene (2.04%) at minute 38.15, respectively (Table 6). There were five (5) unidentified compounds which were also detected in the H_2_O_2_ treated plants under drought stressed conditions.

**Table 6:** Retention time (RT) and relative abundance (%) of the volatile compounds (identified and unknown) in the leaf extracts of drought stressed *F. deltoidea* plants treated and untreated with H_2_O_2_.

**Pk#**	**RT**	**Compound**	**% Content**				
			**T0**	**T1**	**T2**	**T3**	**T4**
1	11.39	U1	2.71	9.32	3.04	1.74	2.30
2	11.92	*(E*)-3-Hexenyl acetate	5.33	0.00	0.00	0.13	0.64
3	14.66	(*E*)-β-Ocimene	0.50	23.08	2.32	71.80	0.00
4	19.02	U2	1.07	0.00	0.00	0.00	2.12
5	21.55	U3	8.62	13.85	13.90	10.93	11.11
6	30.59	U4	12.71	8.22	13.15	8.47	23.94
7	32.48	α-Ylangene	3.38	0.00	0.00	0.00	0.00
8	32.69	α-Copaene	2.37	1.96	5.07	0.58	3.70
9	33.52	β-Elemene	0.00	0.71	0.00	0.00	0.00
10	34.72	(*E*)-Caryophyllene	38.88	15.36	20.21	0.04	16.73
11	35.50	α-*trans*-Bergamotene	5.42	0.00	0.00	0.00	0.00
12	36.28	α-Humulene	12.70	0.54	1.03	0.00	0.64
13	37.52	Germacrene D	1.26	15.86	32.90	3.18	26.50
14	37.75	δ-Selinene	0.00	6.73	0.00	0.00	0.00
15	38.15	α-Guaiene	0.00	1.76	2.04	0.00	0.00
16	38.35	U5	2.12	1.57	3.23	1.01	4.55
17	38.76	(*E,E*)-α-Farnesene	2.94	0.04	0.04	2.13	4.40
18	39.38	δ-Cadinene	0.00	1.01	3.07	0.00	3.38

Pk#, peak number; T0, untreated control; T1, treatment with 5 mM hydrogen peroxide (H_2_O_2_); T2, treatment with 10 mM H_2_O_2_; T3, treatment with 15 mM hydrogen peroxide (H_2_O_2_); T4, treatment with 20 mM H_2_O_2_.

**Figure 10:**
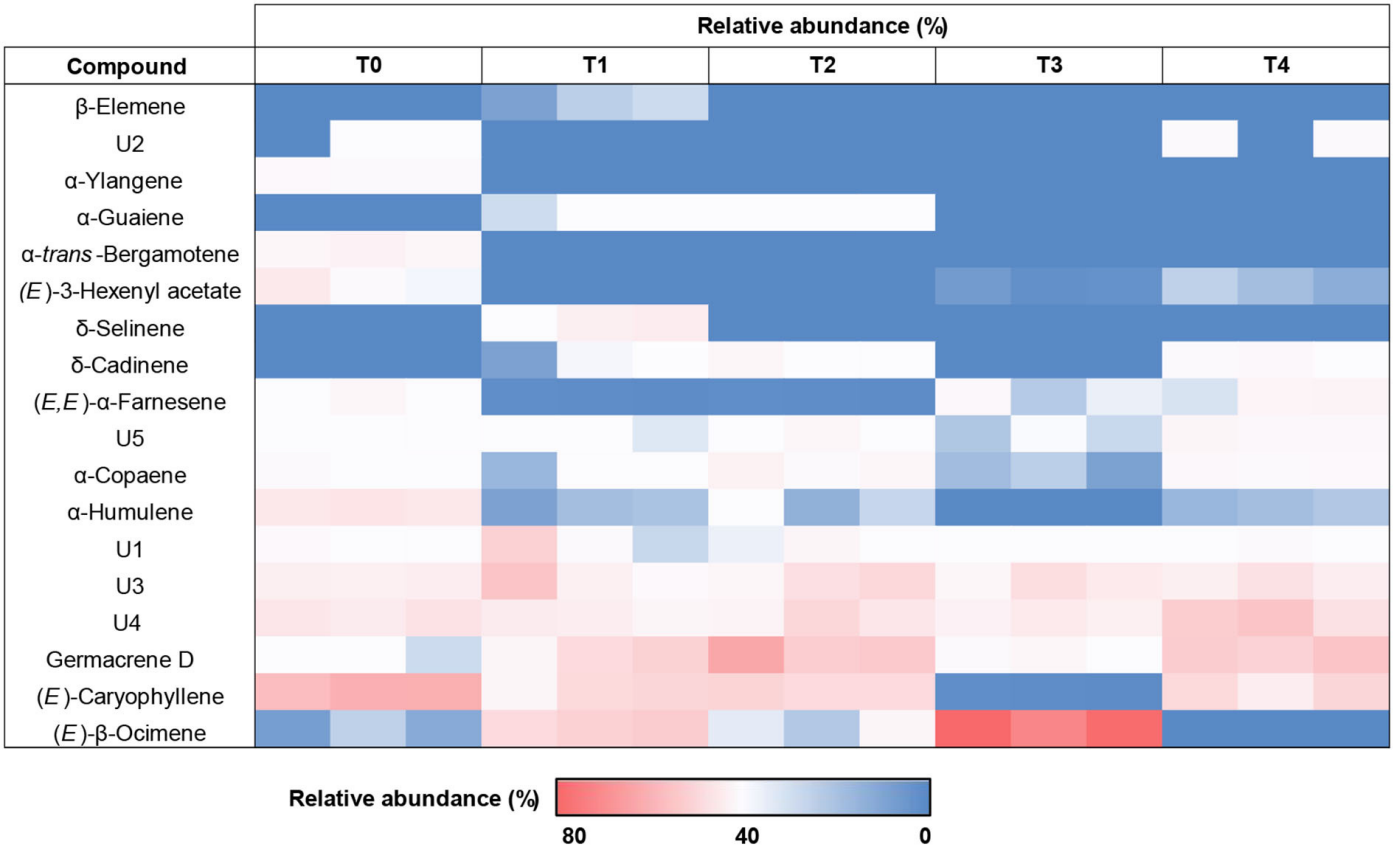
Clustered heat map based on increasing average relative abundance (%) of the volatile compounds (identified and unknown) in the leaf extracts of H_2_O_2_ treated *F. deltoidea* and untreated plants under drought stress.

Heat map clustering analysis showed increasing average percentages of certain compounds (pattern; red > white > blue). These compounds were found to occur, be absent and/ or increase in *F. deltoidea* leaf extracts corresponding to H_2_O_2_ treatments (Figure 10). As a result, a significant increase of (E)-β-Ocimene was clearly shown after the plant underwent T1 (23.08%) and T3 (71.80%) treatments, but the percentage was lessened to trace amounts for the T4 application. Besides, T1 and T2 treatments resulted in a higher abundance of Germacrene D with respective 15.86% and 32.90% compared to the native (control) plant. Following T4, the Germacrene D amount was found to be slightly less (26.50%). T1 also ensued the occurrence of several other volatile compounds including δ-Selinene (6.73%), while T2 resulted in a higher amount of α-Guaiene (2.04%), and δ-Cadinene (3.07%). Unidentified compound abundances also occur more intensely after T1 such as presented by U1 and U3. However, some compounds including (E)-Caryophyllene, α-Humulene, α-Ylangene, and ethyl acetate were found to have decreased. Following T4, δ-Cadinene (3.38%), (E, E)-α-Farnesene (4.40%), and U4 (23.94%) relative abundance were found to be higher than the control plant without treatment.

## Discussion

Drought stress induces negative effects in plants including morphological, physiological, molecular, and metabolic changes which need to be improved by different means for better growth and development. Our results indicate that the number of leaves increased significantly with the application of H_2_O_2_ during drought conditions. This was due to the role of H_2_O_2_ in protein initiation, as well as in plant signaling and development (Barba-Espín et al., 2011; Ralmi et al., 2021). The treatments produced enhancing effects on plant height and leaf area of the *F. deltoidea*, although the results were not significant. H_2_O_2_ plays a role in increasing endogenous levels or to regulate relative gene expression. Liu et al. (2013) reported that the application of H_2_O_2_ positively influenced the plant metabolism and antioxidant enzyme production leading to better plant growth. It can be noted that drought stress leads to a decrease in photosynthetic activity due to stomatal closure. This study proved that the rate of photosynthesis was significantly improved with the application of H_2_O_2_ on stressed *F. deltoidea* plants. H_2_O_2_ is a signaling molecule, which creates a positive response under abiotic stress, such as drought (Ashraf et al., 2015). It is obvious in this study that exogenous H_2_O_2_ enhanced drought resistance by protecting the structures of organelle under drought stress conditions. In addition, H_2_O_2_ protects chloroplast/mesophyll ultra-structure, modulates the expression of the rubisco gene, and increases the actions of antioxidant enzymes to preserve photosynthesis during drought stress conditions (Liao et al., 2012).

H_2_O_2_ application reduces the transpiration rate in stressed *F. deltoidea* plants to some extent, which has been confirmed from this study. The reduced transpiration rate is an indicator of high water-use efficiency. Sofo et al. (2005) reported that H_2_O_2_, being an important signaling molecule, may increase water use efficiency, the recovery of net transpiration rate, and the net photosynthetic rate of plants. Increase in stomatal conductance with the application of H_2_O_2_ is a tool to mitigate drought stress since it helps to control adaptive transpiration as reported by Hessini et al. (2008). Lawlor (1995) stated that when increasing the stress when the stomata is closed the presence of inner leaf CO_2_ also declines. The opening and closing of the stomata is a mechanism in plants which regulates the water loss by sacrificing CO_2_ uptake, especially when the environmental conditions are disparaging as reported by Arve et al. (2011). However, the results obtained from this study opposed these statements, and noted that the spraying of H_2_O_2_ at the concentrations of 15 and 20 mM could significantly improve the CO_2_ content in stressed *F. deltoidea* plants. H_2_O_2_ also plays a vital role in brassinosteroid-regulated stomatal movement (Shi et al., 2015).

As per reports of others (Santos, 2004) under abiotic stresses the leaf chlorophyll content decreases due to an inhibited synthesis of 5-aminolaevulinic acid, but this study indicates a significant increase of chlorophyll content with H_2_O_2_ treatment in the *F. deltoidea* plants. It has been proclaimed by Steffens et al. (2013) that the withdrawal of the negative impacts of stresses through the application of H_2_O_2_ promoted plant chlorophyll content. Improved chlorophyll content might be due to the non-enzymatic defense mechanism especially due to activity of ROS-scavenging proteins under stress conditions. It is also speculated that this might be caused by the role of H_2_O_2_ itself as a signal of oxidative stress since it changed the redox status to antioxidative status of the surrounding cells (Sairam and Srivastava, 2000). Our study indicates that relative variable fluorescence and photosynthetic yield were significantly higher in treated plants under drought field conditions. This higher photosynthetic yield is also an indication of stress removal in the presence of exogenous H_2_O_2_. Our results showed that all the plant growth and physiological parameters were positively correlated with each other in H_2_O_2_ treated *F. deltoidea* plants. Improved plant physiological parameters create significant effects on plant growth and development under drought-stressed conditions.

Carotenoid content of pea seedlings was reduced when they were subjected to drought conditions (Moran et al., 1994). Our results show that carotenoid content was significantly improved with the spraying of 15 and 20 mM of H_2_O_2_ on *F. deltoidea* stressed plants. Total phenolic and flavonoid contents were also significantly improved with the application of H_2_O_2_ in *F. deltoidea* plants. The possible reason for higher phenolic and flavonoid content in treated plants was probably due to the fact that H_2_O_2_ acted as a signaling molecule in phenolic synthesis (Zhang et al., 2013; Jamaludin et al., 2020). Chen et al. (2007) reported that the accumulation of proline, sugars, and polyols are responsible for plant survival under abiotic stresses. Soluble sugars also take part in signaling to regulate molecules in numerous plant growth and developmental processes. Our results demonstrate that the total sugar content of stressed *F. deltoidea* plants was significantly amended with the application of H_2_O_2_. The sucrose phosphate synthase (SPS) activity might be improved due to the regulatory effects of H_2_O_2_, and it regulated the manufacture of sucrose from triose phosphates during photosynthesis (Uchida et al., 2002).

We found that the spraying of H_2_O_2_ improved antioxidant activity in *F. deltoidea* plants under drought stress conditions. H_2_O_2_ may alleviate the level of antioxidants in plants by activating PAL, CHS, and stilbene enzymes (Nyathi and Baker, 2006). Zhang et al. (2001) suggested that under stressed conditions, when guard cells are amended with ABA, it may close the stomata through the production of H_2_O_2_, and H_2_O_2_ plays a role as intermediate in ABA signaling. This is in parallel with our results which indicate that the application of H_2_O_2_ promoted stomatal closure of *F. deltoidea* plants. The stomatal closure during drought stress is also related to the maintenance of xylem integrity (Jones and Sutherland, 1991).

The application of H_2_O_2_ improved the sodium (Na^+^) content in both leaves and syconium under drought stress. The beneficial effects of Na^+^ are the same as nitrogen (${\text{NO}}_{3}^{-}$ and ${\text{NH}}_{4}^{+}$) or K^+^, which regulates plant growth by improving leaf chlorophyll content, net photosynthetic rates, water use efficiency, nitrate reductase activity and relative growth rate (Brownell and Jackman, 1966; Smith et al., 1980; Redondo-Gómez et al., 2010; Gattward et al., 2012). Nitrate reductase enzyme catalyzes the reduction of nitrate N to organic forms and reflects the level of nitrogen (N) activity in plant leaves (Lane et al., 1975). Na^+^ also enhances nitrate uptake by roots and increases assimilation of nitrate in leaves (Ohta et al., 1989). The improved level of Na content may increase the activity of N in plants which stimulates plant growth and development in drought conditions. The photosynthetic capacity of leaves is interconnected with the nitrogen content, predominantly because the proteins of the Calvin cycle and thylakoids exemplify most of the leaf nitrogen (John, 1989). The treatment of 20 mM H_2_O_2_ produced the highest amount of K content in the plant syconium. The elevated level of K is essential in regulating the osmotic potential and water uptake in water-stressed plants. Since H_2_O_2_ is related to stomatal closure, it helps to adjust to drought stress in escalating plant resistance (WHO Regional Office for Europe, 2000). Moreover, the nutrient K, helps in galvanizing different enzyme systems and thereby regulates water movement and water use efficiency, nitrogen uptake and protein building and photosynthesis [Risk Assessment Studies (RAS), 2004]. In a study with broad beans, Kazerouni et al. (2001) observed that potassium improved the water content in the plant leaves, and it increased the tolerance to water stress. Hence, under environmental stress conditions the enhancement of K content of plants is important for the survival of crops (Cakmak, 2005).

The higher amount of Ca is a good indicator of improved plant condition under stressed conditions. The elevated level of Ca^2+^ in a cell acts as a signal carrier and transporter into appropriate biological responses by downstream effectors. In this study, the amount of Ca content in the leaves was found to be greater in 10 mM H_2_O_2_ treated plants. The higher amount of Ca in plants helps to alleviate the adverse effects of drought stress. The exogenous application of Ca^2+^ and H_2_O_2_ also recovered the physiological damage of *Brassica* seedlings under drought stress (Khan et al., 2017). Pei et al. (2000) reported that, H_2_O_2_ production by induction of ABA and the H_2_O_2_-activated Ca^2+^ channels are significant for ABA-induced stomatal closing under stress conditions. Our results indicate that magnesium (Mg) content in the leaves was significantly higher in 15 mM H_2_O_2_ treated plants. The leaf magnesium plays a decisive role in the process of photosynthesis and is one of the major contributing factors of biomass formation (Tränkner et al., 2016). Liu and Porterfield (2014) explained the role of H_2_O_2_ in alleviating the effects of stresses as they demonstrated the use of magnesium peroxide for flooded corn as an effective oxygen buffer, which generated H_2_O_2_ and released biological oxygen for the stressed plants.

Our results indicate that arsenic (As) content was increased in the 10 mM H_2_O_2_ treated leaves while the highest As content in the syconium was present in the control plants. Higher doses of arsenate reduce seed germination, plant biomass accumulation, and produce oxidative damage (Mascher et al., 2002; Shri et al., 2009). But our results showed a positive result in plant growth and development, it was suggested that the application of foliar H_2_O_2_ promoted As uptake but not to a harmful level. The antimony (Sb) content in leaves was the highest in the 20 mM H_2_O_2_ treatment, while the 10 mM H_2_O_2_ treated plants had the highest Sb content in the plant syconium. Kabata and Pendias (2001) suggested that 5–10 mg kg^−1^ of Sb in plant tissue are phytotoxic, but as per reports of Eikmann and Kloke (1993) 5 mg kg^−1^ Sb in plants is at a tolerable level. Pan et al. (2010) also noticed the reduced plant growth and biomass accumulation due to Sb pollution. Thus, although the application of H_2_O_2_ improved Sb uptake, it was not up to its toxicity level in the plants.

Our results indicate that iron (Fe) accumulation was significantly improved in the leaves and syconium with the application of H_2_O_2_. Fe deficiency leads to severe chlorosis and reduction in yield and also the nutritional content of the plants (Marschner, 2002; Curie and Briat, 2003). Additionally, Fe is helpful in many physiological processes such as photosynthesis, plant respiration, sulfate assimilation, synthesis of hormones, nitrogen fixation, DNA synthesis (Hell and Stephan, 2003; Puig et al., 2007). The improved physiological activities of treated plants may be due to the higher level of Fe in the plants. Ravet et al. (2009) demonstrated that ferritin protein in plants is responsible for controlling the levels of ROS, enzymatic activity is involved in ROS detoxification, which is a crucial role for the protection against oxidative damage in plants.

The distribution of stomata was found more frequent on the abaxial surface of leaves in treated *F. deltoidea* plants in comparison to the adaxial surface of the leaves, which is located on the epidermal layer of the leaf. Fu et al. (2013) testified that under drought stress conditions, stomatal density in eggplants at the abaxial surface increased by 20.39% compared to well-watered plants. As per reports of Liu et al. (2009) usually the stomatal numbers and pore aperture numbers would decrease during the periods of water stress, which is a common occurrence in *Arabidopsis* and grass species. Nonetheless, in some plant species it was disclosed that a decreased water level led to an increased stomatal density (Fraser et al., 2009). Moderate drought stress enhances the leaf thickness which may produce more guard cells for a given leaf area and increase stomatal density. Other than that, we suggest that the application of H_2_O_2_ could protect the leaf's margin from damage. This observation might be correlated to the function of H_2_O_2_ as a protective and signaling molecule which mediates the negative effects of drought stress in plants. Asad et al. (1998) suggested that H_2_O_2_ induced safety against lethal and mutagenic effects in *Escherichia coli* cells. Rose and Swift (2014) described the scorching of leaves which could appear as a spot or a burn along the leaf margin under drought stress. Hence, spraying the right amount of H_2_O_2_ could reduce the damage caused by water deficiency as shown in our results.

Thicker palisade parenchyma cells and a higher number of stomata make plants resistant to drought conditions. Moreover, our results indicate that the application of H_2_O_2_ reduced the damage of mesophyll cells in stressed plants by causing thicker mesophyll cells when compared to the untreated plants. In opposition, Sun et al. (2012) stated that tomato plants under mild stress conditions displayed a reduced palisade and spongy tissue thickness. Baum et al. (2000) noticed that abiotic stress affects the thickness of xylem and phloem cells, which regulates the xylem and phloem transport. The application of H_2_O_2_ in this study improved the leaf vein structure under drought stress field conditions. It is expected that the treated plants work better at carbon and water transportation as in plants with an improved structure of phloem and xylem. Nasir et al. (2021) also reported that a lower concentration of H_2_O_2_ improved the tracheary elements and vascular cell of Mas Cotek leaves. Additionally, treated *F. deltoidea* plants might lead to better cross-linking of the cells and tissue of xylem and phloem. Hamann (2012) recorded that the strength and rigidity of the cell walls are improved by the cross-linking process which is an important mechanism in plants to mitigate environmental stresses.

Rubisco is a major enzyme which assimilates atmospheric CO_2_ into the biosphere and is the most imperative target for improving the efficiency of photosynthesis in vascular plants (Parry et al., 2013). The progressive down-regulation of *rubisco* directs to a reduced RuBP content in plants during severe drought thereby inhibiting photosynthetic CO_2_ assimilation (Flexas and Medrano, 2002). Our results indicate that the *Rubisco* gene expression was upregulated because of exogenous H_2_O_2_, and we suggest that it contributed to better adaptation of the *F. deltoidea* var. *deltoidea* plants by increasing photosynthetic rates and photosynthates accumulation under drought-stress. The results also show that the *rbcL* gene expression ratio was the highest in the 15 mM treatment compared to the 20 mM and 0 mM H_2_O_2_ treatments. Better *rbcL* gene expression in the 15 mM H_2_O_2_ treatment may trigger plant growth and development in drought stress conditions. It can be speculated that the exogenous H_2_O_2_ improved the levels of gene expression leading to better relative stability in the plants (Liu et al., 2010). This indication is supported by this study, that the net photosynthetic rate of all H_2_O_2_-treated plants increased in drought stress conditions. H_2_O_2_ as a ROS growth promoting chemicals induced the largest changes in the gene expression levels in various plants and this was probably due to its relative stability (Li et al., 2009).

In this study a total of 18 volatile compounds were detected from H_2_O_2_ treated and untreated *F. deltoidea* plants under drought conditions. These compounds were reported to be responsible for the aroma of *F. deltoidea* as well as other *Ficus* species (Grison-Pigé et al., 2002). Among the unidentified compounds, U4 peak at min 30.59 with a significantly high relative abundance (12.71%) is suggested to be the linalool which is reported as among the major essential oils in *F. deltoidea*. U4 is also less volatile, and thus detected later than ocimene which corresponds to the reported linalool (Grison-Pigé et al., 2002; Lee et al., 2011). The results of heat map clustering analysis showed that the percentage of certain compounds in treated leaves increasingly corresponded to H_2_O_2_ treatment under drought conditions. This finding indicates that treatment of *F. deltoidea* with H_2_O_2_ influences the accumulation of the volatile oils. Such similar occurrence was reported focusing on accumulation of volatile oils mediated by H_2_O_2_ production (Wang et al., 2011).

Drought stress affects synthesis of photosynthetic pigments and protein content, electron transfer chain (ETC), rubisco and sucrose phosphate synthase (SPS) enzyme activity, and the percentage of aromatic or volatile oils of plants (Bakshi et al., 2019). Our results show that exogenous H_2_O_2_ improved plant growth processes, protected cellular structure of leaves from damage, increased mineral accumulation and the level of *Rubisco* gene expression under drought stress. The percentage of volatile oils content in leaves of treated plants increased significantly compared to the control plants. The reduction of volatile oils in the control plants in this study may be due to the disturbance of the photosynthesis process and reduction of photosynthates accumulation in plants under drought stress, which suppresses plant growth and development (Flexas and Medrano, 2002). The disrupted function of the Calvin cycle and depletion of leaf area due to drought stress reduces the biosynthesis of volatile oils. The reduction of volatile oils in plant parts is a consequence of drought stress (Razmjoo et al., 2008). H_2_O_2_ is a non-radicle reactive oxygen species, which due to its stability and diffusion through the plasma membrane acts as a secondary messenger and regulates the flow of Ca^+2^, protein synthesis and gene expression (Bienert et al., 2006).

Volatile oils belong to the group of terpenoids and are synthesized *via* the cytosolic mevalonic acid (MVA) pathway or methylerythritol phosphate (MEP) plasticity pathway (Perreca et al., 2020). Monoterpenes (synthesis by MEP pathway) and Sesquiterpenes (synthesis by MVA pathway) are the main constituents of volatile oils that play a role in synthesis of photosynthetic pigments, aroma, flavor, plant defense system and antioxidant activities (Pavela et al., 2021). H_2_O_2_ elevates the level of expression of saponin synthesis (SS), squalene epoxidase (SE) and β-amyrin synthase (β-AS) enzymes which regulate the biosynthesis of volatile oils (Hu et al., 2002, 2004; Kim et al., 2006). H_2_O_2_ also acts as a signaling molecule which mediates the biosynthesis of saponin in response to oligogalacturonic acid (OGA) and jasmonic acid (JA) (Zhao et al., 2010). In this study, the optimum concentrations of H_2_O_2_ (10–15 mM) may mediate the biosynthesis of volatile oils in *F. deltoidea* plants under drought conditions. The improved level of volatile oils plays a significant role in mitigating the negative effects of drought as well as other abiotic stresses of plants.

## Conclusion

It is concluded that foliar applications of 10–15 mM of H_2_O_2_ should be applied biweekly to increase plant growth, photosynthesis, stomatal conductance, and chlorophyll fluorescence in *F. deltoidea* plants grown in drought stress conditions. In addition, chlorophyll, carotene, total phenols, total flavonoids, sugar content, and antioxidant activities of stressed plants are increased significantly with the H_2_O_2_ treatments leading to sustainable plant growth under drought-stressed conditions. The enhancement of macro nutrients content in the treated plants alleviates the negative effects of drought stress by improving plant physiological activities. A slight increase of arsenic and antimony at safe levels in the treated plants contributes to stress mitigation as well. Furthermore, foliar applications of H_2_O_2_ improved the cellular and vein structure of the leaves and reduced the damage in leaves caused by drought stress. Similarly, the application of 15 mM H_2_O_2_ should be done to up-regulate the expression of the *Rubisco* gene in stressed *F. deltoidea* plants to catalyze the assimilation of CO_2_ and increase net photosynthesis. Foliar application of H_2_O_2_ increased the accumulation of volatile oils in the plants, and the improved level of volatile oils mitigates the negative effects of drought stress. All these effects and influences help to alleviate the undesirable effects of drought stress. Our findings suggest that a foliar application of H_2_O_2_ is a novel technique to reduce the negative effects of drought stress in plants.

## Data availability statement

The original contributions presented in the study are included in the article/supplementary material, further inquiries can be directed to the corresponding author.

## Author contributions

MK: conceptualization, funding acquisition, supervision, data curation, and writing—original draft and editing. RJ: data curation, writing—original draft, and statistical analysis. AM, ZR, and SK: review and language editing. HA-Y, NB, MA, and KM: review, editing, and validation. All authors contributed to the article and approved the submitted version.

## Funding

This research was funded by the Kementerian Pengajian Tinggi (KPT), Fundamental Research Grant Scheme (Project Code: FRGS/2/2014/SG03/UNISZA/02/1).

## Conflict of interest

The authors declare that the research was conducted in the absence of any commercial or financial relationships that could be construed as a potential conflict of interest.

## Publisher's note

All claims expressed in this article are solely those of the authors and do not necessarily represent those of their affiliated organizations, or those of the publisher, the editors and the reviewers. Any product that may be evaluated in this article, or claim that may be made by its manufacturer, is not guaranteed or endorsed by the publisher.
